# Editorial: Through the lens of fear: unsafe neighborhood and mental health difficulties among adults

**DOI:** 10.3389/fpubh.2025.1728352

**Published:** 2025-10-31

**Authors:** Sahar Obeid, Sally Souraya, Marie Anne El-Khoury, Myriam El Khoury-Malhame, Feten Fekih-Romdhane, Souheil Hallit

**Affiliations:** ^1^Department of Psychology and Education, School of Arts and Sciences, Lebanese American University, Beirut, Lebanon; ^2^Royal College of Psychiatrists, London, United Kingdom; ^3^Department of Psychology and Education, School of Arts and Sciences, Lebanese American University, Beirut, Lebanon; ^4^The Tunisian Center of Early Intervention in Psychosis, Department of Psychiatry “Ibn Omrane”, Razi Hospital, Manouba, Tunisia; ^5^Faculty of Medicine of Tunis, Tunis El Manar University, Tunis, Tunisia; ^6^School of Medicine and Medical Sciences, Holy Spirit University of Kaslik, Jounieh, Lebanon; ^7^Department of Psychology, College of Humanities, Effat University, Jeddah, Saudi Arabia; ^8^Applied Science Research Center, Applied Science Private University, Amman, Jordan

**Keywords:** unsafety, neighborhood, mental health, fear of unsafety, adults

Neighborhood safety is increasingly recognized as a critical determinant of both mental and physical wellbeing (1). Beyond basic physiological needs, a sense of safety is fundamental for psychological stability, social engagement, and self-actualization (2, 3). Unsafe environments—whether characterized by crime, violence, or social disorder—are consistently linked to heightened stress, depressive symptoms, anxiety, reduced physical activity, and even adverse birth outcomes (4–6). While much research has focused on Western urban populations, this Research Topic brings studies that broaden our understanding of neighborhood safety across diverse cultural, socioeconomic, and geographic contexts, shedding light on both risk and protective factors.

A central theme emerging from this Research Topic concerns the conceptualization and measurement of perceived safety. The study titled “*Psychometric properties of the Feeling of Unsafety Scale – Arabic in general population adults*” offers a rigorously validated instrument for assessing perceptions of neighborhood unsafety among Arabic-speaking adults (Obeid et al.). This psychometric contribution is crucial for ensuring the precise assessment of subjective experiences, particularly in contexts where official data may be incomplete, inconsistent, or unreliable. By providing a culturally and linguistically adapted measure, the study establishes a methodological foundation for future research aiming to elucidate the association between perceived safety and both mental and physical health outcomes.

Another significant contribution explores the multifaceted relationships between mental health and social determinants. The cross-sectional study on loneliness and depression among men in Poland elucidates how social isolation, compounded by environmental stressors, contributes to variations in psychological wellbeing (Dziedzic et al.). Similarly, research examining urban-rural disparities in women's mental health demonstrates that neighborhood characteristics—such as social cohesion, accessibility of services, and exposure to environmental stressors—play a critical role in shaping vulnerability to depression and anxiety (Chen et al.). Taken together, these studies underscore that perceptions of safety extend beyond the mere presence of crime or violence, reflecting instead the broader social milieu, community dynamics, and demographic factors that structure individuals' lived experiences.

The influence of neighborhood environments on physical health is also well-documented. An analysis of Chinese resident's health based on data from the CGSS 2021 reveals that the interaction between neighborhood conditions and social networks significantly affects overall wellbeing (Wang et al.). Furthermore, research on psychosis in Trinidad underscores how exposure to neighborhood-level violence can aggravate severe mental health conditions (Roberts et al.). Collectively, these findings provide additional evidence of spatially patterned health risks and highlight the critical importance of geographically targeted public health interventions.

Across this body of research, several convergent themes emerge. First, perceived neighborhood safety represents a multidimensional construct influenced by both objective environmental threats and the social and cultural contexts in which individuals are embedded. Second, unsafe neighborhood conditions exert measurable effects on mental health—manifesting in heightened stress, depressive symptoms, and, in some cases, severe psychiatric outcomes—while simultaneously compromising physical wellbeing through stress-related psychological pathways and constraints on health-promoting behaviors. Third, protective factors such as social cohesion, community support, and equitable access to resources appear to mitigate these detrimental effects, underscoring critical targets for prevention and intervention efforts.

The Research Topic further highlights the imperative of broadening neighborhood safety research beyond Western urban contexts. By encompassing diverse populations—including Arabic speaking adults, rural and urban women across different nations, and communities in low-and middle-income regions—these studies provide culturally nuanced and globally applicable insights. Collectively, they emphasize the necessity of interdisciplinary collaboration across psychology, public health, urban planning, and social policy to comprehensively address the multifaceted relationships between environmental safety and human wellbeing.

Looking ahead, this body of work identifies several promising directions for future research. Longitudinal designs are essential to disentangle the causal mechanisms linking perceived safety, social context, and health outcomes. Additionally, intervention-based research should examine evidence-based strategies aimed at enhancing perceptions of neighborhood safety, fostering social cohesion, and alleviating the effects of environmental stressors. The resulting evidence can inform policymakers and practitioners in developing community-level initiatives that not only reduce objective risks but also enhance residents' subjective sense of security, ultimately promoting improved mental and physical health.

In conclusion, the studies featured in this Research Topic collectively deepen our understanding of how neighborhood safety—or its lack—affects human wellbeing across varied populations and contexts. By emphasizing advancements in measurement, elucidating psychosocial mechanisms, and exploring environmental interactions, this Research Topic highlights the critical need to incorporate neighborhood safety into public health strategies and mental health interventions. As the world continues to face challenges related to urbanization, conflict, and social inequalities, creating safe and supportive environments will be essential for fostering holistic health and resilience.
